# Outsmarting SARS-CoV-2 by empowering a decoy ACE2

**DOI:** 10.1038/s41392-020-00370-w

**Published:** 2020-11-03

**Authors:** Milena Sokolowska

**Affiliations:** 1 Swiss Institute of Allergy and Asthma Research (SIAF), University of Zurich, Davos, Switzerland; 2 Christine Kühne - Center for Allergy Research and Education (CK-CARE), Davos, Switzerland

**Keywords:** Molecular medicine, Structural biology, Vaccines

Along with the current efforts to develop high-affinity neutralizing antibodies, Chan and colleagues engineered the soluble variant of human ACE2 with enhanced binding to the spike protein, outranking the soluble wild-type protein in blocking SARS-CoV-2 infection in vitro. In addition, the newly created soluble ACE2 is enzymatically active in cleaving angiotensin-2, increasing its therapeutic potential in COVID-19.^1^

**Fig. 1:**
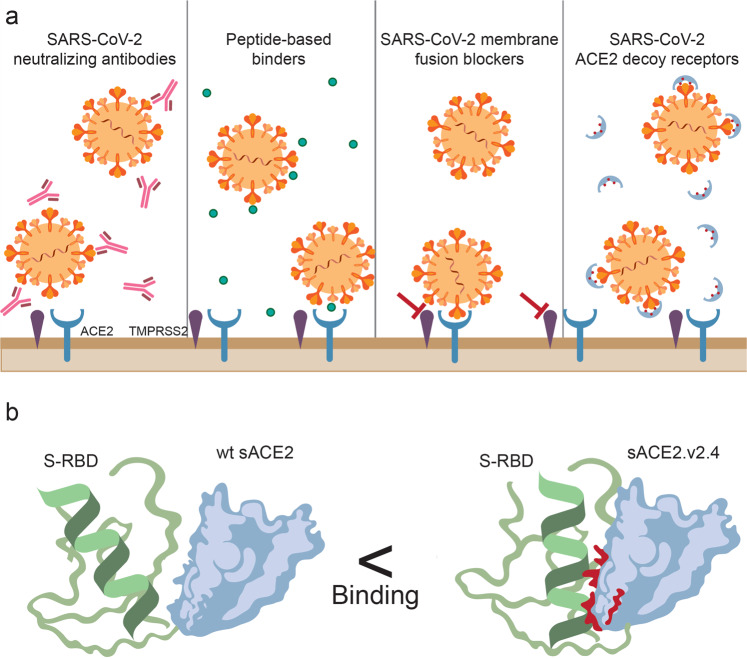
Current approaches to neutralize SARS-CoV-2 spike protein and to prevent its binding to the cellular ACE2. **a** Cocktails of neutralizing antibodies against spike protein of SARS-CoV-2, peptide-based binders blocking SARS-CoV-2-RBD-ACE2 interface, small molecule inhibitors blocking the host cell proteases, and soluble recombinant human ACE2 are currently being under different stages of development against COVID-19. **b** Chan and colleagues engineered a stable soluble variant of ACE2 with enhanced binding affinity to the RBD of SARS-CoV-2 spike protein, sustained angiotensin-2 peptidase activity, and superior abilities to block SARS-CoV-2 infection in vitro. RBD receptor-binding domain. Created by Zuzanna Lukasik, MD

In times of COVID-19 death toll exceeding one million cases and facing devastating social and economic repercussions, the whole world is awaiting development of a vaccine and potent treatment options. There are currently a few therapeutic approaches focused on blocking SARS-CoV-2 binding to its key receptor, an angiotensin-converting enzyme 2 (ACE2), or on inhibition of virus spike cleavage (Fig. 1a). These approaches potentially prevent the primary infection or block the spread of the virus. The most advanced at the moment seems to be the antibody-based therapies, including convalescent plasma-based therapy and cocktails of neutralizing antibodies. However, SARS-CoV-2 S protein might develop accumulating mutations to escape the selection pressure.^2^ Therefore, other approaches are also intensively studied including soluble recombinant human ACE2 (rhACE2) or peptide-based binders, developed to block SARS-CoV-2-RBD-ACE2 binding interface or small molecule inhibitors blocking the host cell proteases, such as TMPRSS2 or furin, block virus fusion with the host cell.^3^

The idea of using soluble ACE2 to prevent acute lung injury (ALI) originated from the first SARS-CoV epidemics. Kenji Kuba with colleagues from Penninger lab showed that the infection of mice with SARS-CoV resulted in decreased expression of ACE2 in the lung, dysregulation of renin–angiotensin system (RAS), leading to ALI.^4^ Thus, delivery of the soluble ACE2 has a potential to prevent or slow down the infection and to protect from ALI by increasing angiotensin-2 proteolysis. The group of Eric Procko followed this idea and initiated the study also rooted in the fact that ACE2 mainly acts as a peptidase, cleaving angiotensin-2, and delivering vasodilating peptides.^1^ ACE2 has never been a “designed” receptor for any coronaviruses. These pathogens evolved to utilize ACE2 of several species to get inside the host cells, using spike protein binding to ACE2, independently of its peptidase domain. Therefore, binding SARS-CoV-2-RBD to ACE2 still could be strengthened by mutations which increase the affinity. Only this time, it should work against the virus by “upgrading” the decoy, soluble ACE2, which then could block SARS-CoV-2 before it binds to cellular ACE2.

Therefore, Kui Chan and colleagues from Procko lab created an ACE2 library containing all single amino acid substitutions at 117 sites at the interface binding to the S protein and the angiotensin-peptide binding cavity.^1^ ACE2 library was expressed in human Expi293F cells, further incubated with spike RBD, fused to GFP, which enabled FACS sorting and subsequent sequencing of genetic material from cells carrying ACE2 variants of high or low binding to RBD. Then, in an approach called deep mutagenesis, the frequencies of ACE2 variants in the transcripts of the sorted populations were compared to their frequencies in the naive plasmid library, allowing for the calculation of enrichment ratios and conservation scores for each amino acid substitution in S-RBD-high and low-binding ACE2 variants. Subsequent mapping of the experimental results revealed that there are two ACE2 residues, N 90 and T92, on the periphery of ACE2-S-RBD binding interface, which are the hot spots for enriched mutations. In contrast, residues buried in the ACE2-S-RBD interface tended to be more conserved. Interestingly, all amino acid substitutions in these two residues, except conserved T92S maintaining the N-glycan, were found to be favorable for binding S-RBD. Next, the authors analyzed the molecular basis of the mutation-enhanced affinity of ACE2-S-RBD binding, using the cryo-EM structure, showing that indeed substitutions at T27, D30E, K31, and K417 of the ACE2 interface should facilitate binding to S-RBD. The authors validated 30 of the mutations identified by the deep mutational scan. They expressed each single substitution mutant and analyzed ACE2-S-RBD binding as previously. It revealed that single substitutions were weak, each increasing ACE2-S-RBD binding not more than two-fold. Therefore, in designing the soluble ACE2 (sACE2) variant the authors introduced the combinations of 3–7 mutations, which gave the large increases in S binding. Then, they chose one variant, sACE2.v2 for purification and further verification. This variant containing four mutations bound stronger to the full-length S protein that the wild-type (wt) ACE2 and outcompeted ACE2-IgG1 for binding S protein. sACE2.v2 successfully competed with anti-RBD antibodies in the sera from three COVID-19 convalescent patients, whereas wtACE2 did not. sACE2.v2 showed 65-fold higher affinity than the soluble wtACE2. Then, this construct was further enhanced, by introducing 3 out of 4 mutations, T27Y, L79T, N330Y, which increased the yield with sustained high S binding. It was followed by lengthening the construct, producing a dimeric stable sACE2.v.2.4, which also effectively competed with serum IgG from convalescent COVID-19 patients and presented affinity in picomolar range, as compared to nanomolar ranges for the soluble wtACE2 (Fig. 1b). Monomeric and dimeric form of engineered sACE2 largely exceeded the potency of wtACE2 to neutralize SARS-CoV-2 and SARS-CoV infection in VeroE6 cells. Importantly, this variant showed slightly reduced, but sustained capacity to cleave angiotensin-2, while effectively blocking SARS-CoV-2 from infecting human cells, thus significantly broadening its therapeutic potential.

Engineered soluble construct of ACE2 with enhanced affinity to SARS-CoV-2 spike RBD opens up several possibilities of its usage in the current pandemics. First, it could be used for determination of the most neutralizing antibodies in the convalescent plasma to increase predictability of plasma-based therapies, at the same time decreasing the risk of unsuccessful, but dangerous therapy. Next, after determining its efficacy in in vivo SARS-CoV-2 preclinical models, it could enter clinical trials. Its potential to elicit an immunogenic response (immunogenicity), its safety and efficacy in COVID-19 would have to be carefully evaluated in various phases of clinical development. Immune response to recombinant human proteins may lead to development of neutralizing antibodies and specific T cell responses, compromising their safety and therapeutic potential. Luckily, already existing data of phase II on safety of the other recombinant human soluble ACE2 (GSK2586881) in patients with acute respiratory distress syndrome (ARDS) (ClinicalTrials.gov: NCT01597635) and pulmonary arterial hypertension (NCT03177603) support the premise of its efficacy. Unfortunately, the development of GSK2586881 in ARDS was prematurely terminated. Hopefully already initiated phase II clinical trial with the same recombinant human ACE2 in COVID-19 patients (NCT04335136) will deliver more promising data in the near future. sACE2 with enhanced affinity to SARS-CoV-2 spike RBD would thus have a double impact in the fight against COVID-19, being able to stop the spread of the virus and actively prevent respiratory failure. Thus, the effective approach shown by Procko lab should be extensively explored in future clinical trials, together with further approaches targeting other proteins implicated in the pathogenesis of SARS-CoV-2,^5^ development of neutralizing antibodies or the race to create the vaccine. Our history, human expansion in the natural environment and subsequent evolution of zoonoses sadly should urge us to develop the broader portfolio of possible therapeutic approaches.
